# Microglia heterogeneity and therapeutic strategies in Parkinson’s disease

**DOI:** 10.3389/fimmu.2026.1739341

**Published:** 2026-02-05

**Authors:** Jing Tian, Xuehui Liu, Zhuoqun Wang, Xue Shi, Chunlei Dai, Li Yang

**Affiliations:** 1 Department of Neurology, The First Automotive Works (FAW) Hospital, Changchun, Jilin, China; 2 Department of Infection Control Office, The First Automotive Works (FAW) Hospital, Changchun, Jilin, China; 3 Department of General Surgery, The First Automotive Works (FAW) Hospital, Changchun, Jilin, China; 4 Department of Gastroenterology, The First Automotive Works (FAW) Hospital, Changchun, Jilin, China

**Keywords:** heterogeneity, immunotherapy, microglia, neuroinflammation, PD

## Abstract

Parkinson’s disease (PD) is the second most common neurodegenerative disorder, characterized by the progressive loss of dopaminergic neurons in the substantia nigra (SN) and the abnormal aggregation of α-synuclein (α-syn). PD exhibits features of a chronic inflammatory disease, significantly affecting peripheral organs and the central nervous system (CNS). Clinical signs include motor symptoms such as rigidity, bradykinesia, and tremor, as well as non-motor symptoms such as psychological and cognitive issues. Microglia are resident immune cells of the CNS, exhibiting high heterogeneity and playing a crucial role in the neuronal degeneration and inflammation associated with PD. In PD, microglia play dual roles: maintaining PD homeostasis by phagocytosing and clearing α-syn aggregates while simultaneously becoming dysfunctional due to aggregate overload. This dysfunction drives their transition to a pro-inflammatory phenotype, exacerbating neurotoxicity. Recently, technological advances like single-cell transcriptomics have revealed the diverse functions and changing phenotypic lineages of microglia in PD, providing new insights into their mechanisms. This review systematically describes the biological traits of microglia and their functional, spatial, genetic, and gender-related differences in PD neurodegeneration. It summarizes new intervention and treatment strategies targeting microglia, highlights recent progress and challenges in preclinical research and clinical trials, and offers guidance for developing precision therapies for PD focused on modulating microglial function.

## 1 Introduction

PD is a neurodegenerative disease affecting the CNS. Its global burden has steadily increased. Epidemiological data indicate that from 1990 to 2021, the global PD population surged by 273.9%. With accelerating population aging, the risk of developing PD will rise significantly (1). PD affects multiple systems, involving protein homeostasis imbalances such as pathological aggregates of amyloid-β and misfolded α-syn, tau protein deposition in the CNS, as well as mitochondrial dysfunction and neuroinflammation (2). The clinical manifestations of PD encompass both motor and non-motor aspects (3–6). Current treatment options for PD primarily include medication, deep brain stimulation (DBS), stem cell transplantation, neuronal repair, gene-targeted therapy, and immunotherapy (7, 8). Levodopa is the gold standard first-line treatment, but it only alleviates motor symptoms, the efficacy diminishing as the disease progresses (9). DBS devices are suitable for patients in the middle to late stages of PD, regulating neural circuit function through electrical stimulation to improve motor symptoms (10). However, neuronal damage during treatment may pose a risk of delayed cognitive decline (11). Gene therapy has demonstrated efficacy in reversing motor symptoms of PD in animal models, but the safety and efficacy of these strategies remain to be tested (12). Therefore, developing treatments capable of halting disease progression and providing neuroprotection represents a major challenge in the current field of PD research.

Microglia are the resident immune cells of the CNS, playing crucial immune, surveillance, and protective roles in maintaining brain homeostasis, together with neurodevelopmental disorders (13). The innate immune response mediated by microglia represents a central link that spans and integrates multiple mechanisms of PD (14). Microglia constitute a heterogeneous population of cells. Under physiological conditions, microglia regulate brain development, shape neural architecture, participate in synaptic pruning, and maintain normal neuronal function by interacting with synapses (15, 16). Under pathological conditions, α-syn aggregation and peripheral immune cell infiltration activate microglia, aggravating neuroinflammation and neuronal damage, thereby accelerating PD progression (17). Due to the heterogeneity of microglia, the pro-inflammatory/anti-inflammatory (M1/M2) dichotomy fails to adequately reflect the complex phenotypes and functions of microglia (18). The application of technologies such as single-cell RNA sequencing (scRNA-seq) has revealed novel activation states of microglia, including Disease-Associated Microglia (DAM) (19), expanding the understanding of the functional continuum of microglia, offering new insights into their dynamic regulatory mechanisms (20).

**Figure 1:**
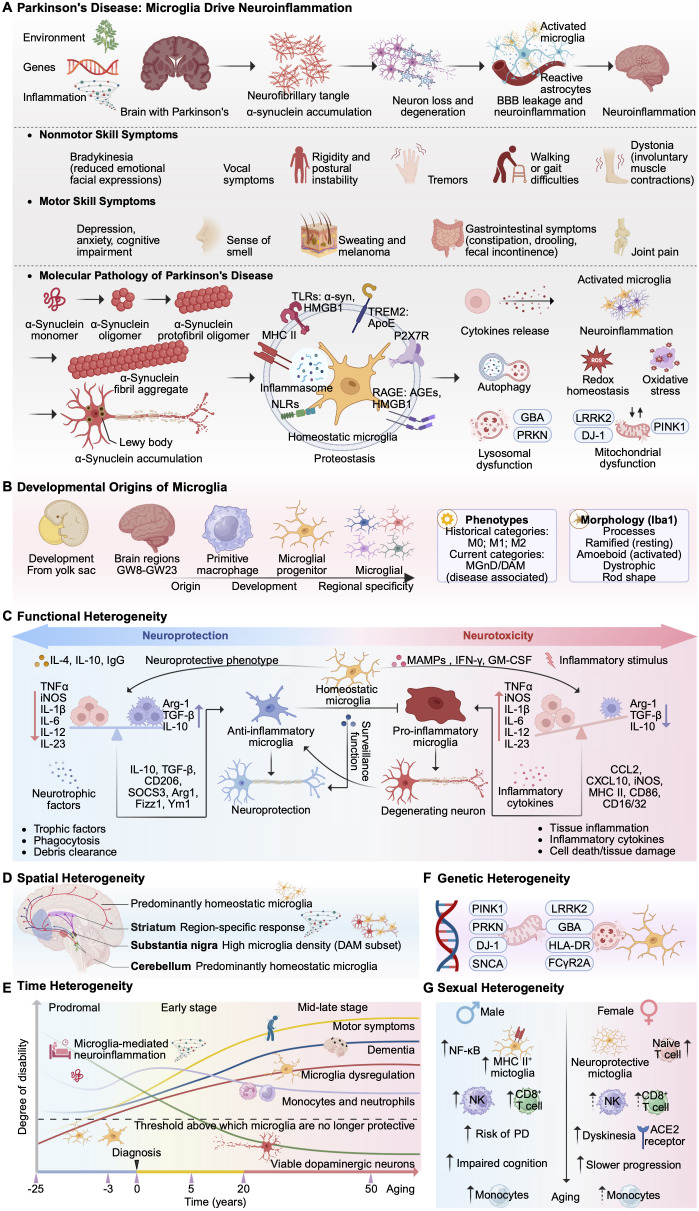
Multidimensional heterogeneity of microglia in Parkinson’s disease. **(A)** Pathogenesis of PD, encompassing etiological triggers (environmental, genetic, and inflammatory), progression of motor/non-motor symptoms, and molecular pathology focusing on α-synuclein aggregation and microglia-driven neuroinflammation. **(B)** Developmental origins of microglia, from yolk sac-derived pro-macrophages to microglial progenitor cells, regional specificity, and morphological phenotypes. **(C)** Glial functional heterogeneity, contrasting neuroprotective versus neurotoxic glial phenotypes, associated signaling pathways, and functions. **(D)** Glial spatial heterogeneity, highlighting region-specific glial responses in brain structures including the striatum, substantia nigra, and cerebellum. **(E)** Temporal heterogeneity of microglia, tracing dysregulation and neuronal loss from the precursor stage through mid-to-late stages of PD progression. **(F)** Genetic heterogeneity of microglia. **(G)** Gender heterogeneity of microglia. ACE2, Angiotensin-Converting Enzyme 2; AGEs, Advanced Glycation End products; Arg-1, Arginase-1; ApoE, Apolipoprotein E; CCL2, C-C Motif Chemokine Ligand 2; Cluster of Differentiation 16/32; CD86, CD16/32, Cluster of Differentiation 86; CXCL10, C-X-C Motif Chemokine Ligand 10; DAM, Disease-Associated Microglia; FCγR2A, Fc gamma Receptor IIA; GBA, Glucocerebrosidase; GM-CSF, Granulocyte-Macrophage Colony-Stimulating Factor; GW, Gestational Weeks; HMGB1, High Mobility Group Box 1; HLA-DR, Human Leukocyte Antigen - DR; iNOS, Inducible Nitric Oxide Synthase; IgG, Immunoglobulin G; IFN-γ, Interferon gamma; IL, Interleukin; LRRK2, Leucine-Rich Repeat Kinase 2; MAMPs, Microbe-Associated Molecular Patterns; MHC, Major Histocompatibility Complex; MGnD, Microglial Neurodegenerative Phenotype; NF-κB, Nuclear Factor kappa-B; PRKN, Parkin RBR E3 Ubiquitin Protein Ligase; PINK1, PTEN-Induced Kinase 1; DJ-1, Parkinson’s Disease Protein 7; RAGE, Receptor for Advanced Glycation End products; SOD2, Superoxide Dismutase 2; TGF-β, Transforming Growth Factor-beta; TLR, Toll-Like Receptor; TREM2, Triggering Receptor Expressed on Myeloid cells 2; TNFα, Tumor Necrosis Factor alpha; α-syn, α-synuclein.

Based on the complexity and plasticity of microglial function, enhancing microglial phagocytosis, reducing neuroinflammation, and promoting their conversion to a protective phenotype represent promising therapeutic strategies for PD (**Figure 1A**). This review systematically outlines recent advances in the paradigm shift of microglia from a dichotomous to a multifaceted, dynamic model. It focuses on the regulatory mechanisms, heterogeneity, and translational research progress of microglia as therapeutic targets, providing a theoretical reference for developing immunomodulatory therapies for PD.

## 2 Functional heterogeneity of PD microglia

Microglia originate from myeloid precursor cells in the vitelline membrane tissue. Through migration, colonization, and differentiation, microglia mature and stabilize within the brain, maintaining the immune homeostasis of the CNS environment (21). As resident immune sentinels of the CNS, the functional state of microglia profoundly influences the progression of PD (22, 23). An intimate understanding of the role of microglia in PD contributes to a deeper comprehension of the disease’s pathogenesis and potential therapeutic approaches (**Figure 1B**).

### 2.1 Functions of microglia in PD

Microglia are resident immune cells of the CNS (24–27). In physiological states, microglia exhibit a highly dynamic dendritic morphology, performing functions such as immune surveillance, waste clearance, and maintenance of synaptic homeostasis, thereby supporting brain development and regulating learning and memory capabilities (28, 29). Microglia express a vibrant array of receptors, including pattern recognition receptors (Toll-like receptors (TLRs)), etc.), phagocytic receptors (TAM (Tyro3, Axl, Mer) receptors, etc.), immunoregulatory receptors (Triggering Receptor Expressed on Myeloid cells 2 (TREM2), CD33, etc.), and multiple neurotransmitter receptors, which respond sensitively to signals ranging from neuronal activity to tissue injury (30–33). Once pathological stimuli are detected, microglia rapidly transition from a ramified to an amoeboid activated state, shifting from sentinels to executors of phagocytosis and inflammatory responses (34, 35).

In the pathological progression of PD, the immune response within the CNS serves as a core driver of neurodegeneration (36). Microglia play a dual role, and the dynamic equilibrium of their functions determines the direction of neuroinflammation in PD. In 1988, the presence of HLA-DR-positive reactive microglia in postmortem tissues of PD patients first linked neuroinflammation to the pathology of PD (37). On one hand, microglia exhibit neuroprotective functions. Through mechanisms such as Toll-like receptor (TLR) 2, TLR4-NF-κB, and L-cystine-associated endocytosis (LANDO), microglia phagocytose and degrade misfolded and aggregated α-synuclein, leading to the clearance of pathogenic aggregates (38). Research has revealed that microglia actively clear accumulated α-syn and tau pathological proteins within neurons by establishing tunnel nanotubes (TNTs). They share healthy mitochondria with neurons, reversing oxidative stress and functional impairment, which performs a crucial neuroprotective function (39). On the other hand, microglia also exert neurotoxic effects. Widespread microglia proliferation is observed in the SN of PD’s brain (4, 40). Misfolded α-syn excessively activates microglia by binding to specific surface receptors (e.g., TLR) or intracellular receptors (e.g., NLRP3), leading to the release of large amounts of proinflammatory cytokines such as IL-1β and IL-6, thereby exacerbating damage to dopaminergic neurons (41). Microglia phagocytose excessively aggregated α-syn, converting it into PD-like lesions, presenting α-syn-associated antigens, recruiting peripheral immune cells to form immune crosstalk, which promotes the malignant progression of PD (42, 43). Activated microglia secrete cytokines (e.g., IFN-γ) that impair lysosomal function in dopaminergic neurons (44). Since microglia are not a homogeneous population, the molecular mechanisms underlying their distinct states and their functional differences in development, aging, and disease are worthy of further investigation.

### 2.2 Functional heterogeneity of microglia

The functional heterogeneity of microglia is closely associated with their highly dynamic morphology and plasticity (**Figure 1C**). During the pathological progression of PD, microglia respond to pathological stimuli through specific activation phenotypes and functional adjustments, with the classic activation phenotype characterized by a dynamic equilibrium between M1 and M2 states, a terminology that has been widely used in early studies (45). However, current research favors state - based classification systems for microglia. The activation of the M1 phenotype leads to the massive secretion of proinflammatory cytokines such as IL-1β and TNF-α, which not only prevents further damage to the central nervous system but may also exert neurotoxic effects on neurons (36, 46). M2 phenotype activation releases anti-inflammatory factors, like IL-10 and TGF-β, along with neurotrophic factors, suppressing excessive inflammatory responses and promoting tissue repair and regeneration, providing neuroprotection for dopaminergic neurons (47). The microglial phenotype exhibits high heterogeneity and dynamism, with activation states forming a continuum rather than discrete polarized states (48). Research has systematically revealed the functional heterogeneity of microglia, defining 12 functionally specialized subpopulations (49, 50). DAM, the microglial neurodegenerative phenotype (MGnD), and proliferative area-associated microglia (PAM), etc., are important microglial subpopulations in PD (51–53). DAM represents one of the most distinct reactive subpopulations, characterized by genes such as ApoE, LPL, and TREM2 that are significantly associated with the progression of neurodegenerative diseases (54, 55).

Behind the functional continuum lies a complex molecular regulatory network. In PD patients, TLRs are activated by pathological α-syn (56). Dectin-1 and TLR4 exhibit synergistic signaling crosstalk that amplifies neuroinflammatory responses. Laminin inhibits Dectin-1, promotes the phenotypic shift of microglia from a pro-inflammatory functional state to an anti-inflammatory and suppresses their neurotoxic effects (57). Neuroinflammation driven by NLRP3 inflammasome activation constitutes a critical component of PD pathological progression, and α-syn induces NLRP3 inflammasome-mediated IL-1β secretion in primary human microglia (58, 59). Reduced nicotinamide adenine dinucleotide phosphate (NADPH) can act as an endogenous inhibitor of P2X7R, alleviating NLRP3 inflammasome activation in microglia to exert neuroprotective effects (60). Different forms of α-syn exhibit distinct properties in activating microglial NLRP3 inflammasomes. Even when polarized toward a proinflammatory phenotype, they may retain protective functions, suggesting that targeting NLRP3 holds potential as a therapeutic strategy for PD (61). At specific stages of PD pathology, microglia exert protective functions. Anti-inflammatory cytokines such as IL-10 can restore microglial homeostasis, enhance phagocytic and clearance activities, and mitigate PD neuropathology (62). The drawback of conventional methods using agonists like capsaicin to stimulate TRPV1 is uncontrolled activation, which can easily lead to excessive Ca²^+^ influx. Instead of protecting microglia, this damages them and triggers harmful inflammatory responses. Research utilizing photothermal nanotechnology enables CS-AT nanoparticles to transiently and controllably activate TRPV1 ion channels on the surface of microglia. This induces moderate Ca²^+^ influx, enhancing the microglia’s autophagy and degradation of α-syn, avoiding the side effects associated with excessive microglial activation (63).

Microglia differentiate into a spectrum of functional states ranging from neurotoxicity to neuroprotection in response to microenvironmental signals. Understanding and targeting the complex neuroinflammatory network mediated by microglia and exacerbated by peripheral immunity, while suppressing immune crosstalk, represents a promising therapeutic strategy for PD.

## 3 Spatiotemporal heterogeneity of microglia in PD

During organismal development, microglia perform multiple tasks, including immune and non-immune functions. Recent advances in functional genomics have revealed the spatiotemporal dynamics of microglial reactivity across various disease contexts (52). The presence and function of microglia subpopulations at different developmental and adult stages, in different brain regions, represent a key direction for investigating their role in PD regulation.

### 3.1 Spatial heterogeneity of microglia

Microglia perform distinct functions at different developmental and adult stages or within specific brain regions. Multiple independent studies confirm that microglia exhibit spatial heterogeneity, displaying distinct gene expression profiles in a region-specific manner (**Figure 1D**). Different microglial transcript clusters are enriched in distinct brain areas of mice, potentially predetermining unique immune responses to pathological irritations in different brain regions (64). Different regions of the human brain harbor distinct microglia subpopulations. Microglia subclusters enriched in the subventricular zone and thalamus exhibit heightened activity, while cerebellar microglia possess unique protein expression profiles (65). Additional studies have confirmed that in the striatum of PD patients, microglia exhibit distinct regional differences compared to other brain regions (66). Furthermore, microglia in white matter express higher levels of antigen-presenting molecules (e.g., HLA-DR) than those in gray matter (67). During the PD process, specific microglia subpopulations may exhibit distinct distributions or responses across different brain regions. Areas with severe α-syn pathology deposition (e.g., SN, striatum) show a higher proportion of DAM, whereas relatively unaffected regions predominantly harbor homeostatic microglia (67). The SN exhibits the highest microglia density in the entire brain, presenting a unique microglia microenvironment where microglia maintain a state of heightened immune vigilance (68). Widespread activation of microglia in the substantia nigra leads to sustained release of cytotoxic factors such as ROS, inducing higher levels of α-syn production and thereby accelerating the progression of PD (68, 69). Microglia exhibit region-specific transcriptional profiles throughout the adult lifespan. The loss of specific immune phenotypes in forebrain regions such as the hippocampus during aging provides a potential cellular basis for explaining the spatially specific pathogenesis of PD pathology (34). Transcriptional heterogeneity in human microglia varies across brain regions and during aging (70). The regional diversity of microglia may also influence regional immune responses. Midbrain microglia located in the lesion core exhibit a distinct anti-inflammatory and phagocytic phenotype, whereas striatal microglia display a pro-inflammatory state, suggesting that potential immunomodulatory targets for microglia may vary across brain regions (71).

The spatial heterogeneity of microglia is a key factor shaping region-specific pathology and neuronal susceptibility in PD. Microglia may perform specific functions adapted to the microenvironment of their brain region. Understanding the regional specificity and temporal dynamics of microglial responses is crucial for elucidating selective susceptibility and disease progression in PD.

### 3.2 Temporal heterogeneity of microglia

Microglia begin migrating into the brain from embryonic day 8.5 and continue until the blood-brain barrier forms. Self-renewal becomes the sole source of new microglia in the healthy brain throughout subsequent life (26). Microglia in embryonic, juvenile, and adult stages exhibit distinct transcriptional profiles, clustering into separate cell groups, indicating that microglia dynamically adapt to the brain’s developmental state (64). The functional state of microglia exhibits temporal heterogeneity in regulating α-syn propagation. Microglia in a resting state limit pathological protein diffusion, whereas their excessive activation or significant loss markedly accelerates α-syn propagation (72). Disturbances in the microglia environment during development may disrupt developmental timing, leading to misregulated expression of inflammatory and other gene pathways and impairing neuronal development (73). Early-onset PD-associated α-syn is more susceptible to triggering microglia polarization toward a proinflammatory phenotype (74). Epigenetic factors Hdac1/2 impair microglia maturation when lost during development but enhance protective functions in disease when lost in adulthood.

The heterogeneity of microglia exhibits age-dependent characteristics, with the functional state of microglia in the brain deteriorating with age (**Figure 1E**) (75). Most adult microglia expressing steady-state genes exhibit high transcriptional similarity, while early postnatal microglia show greater heterogeneity (76). In human microglia, gene expression associated with motility and cell migration decreases with age, consistent with the reduced motility observed in microglia from aged mice (77). The most significant non-genetic risk factor for PD is aging (78, 79). All microglia are affected by aging, and microglia exhibit further transcriptional alterations in PD (80). Age-related inflammatory changes exert a dual impact on the pathogenesis of PD. An ongoing low-grade neuroinflammatory environment creates conditions conducive to pathological aggregation of α-syn. Age-related inflammatory changes exert a dual impact on the pathogenesis of PD. An ongoing low-grade neuroinflammatory environment creates conditions conducive to pathological aggregation of α-syn (81). Concurrently, aging microglia exhibit impaired clearance functions, rendering them incapable of effectively degrading abnormal proteins. These two mechanisms collectively heighten the vulnerability of dopaminergic neurons, contributing significantly to the initiation and progression of PD. A distinct subpopulation of pro-inflammatory and interferon-responsive microglia emerges in the aging brain (82). Stress-induced aging microglia are rich in DAM features (83). Research has found that the proportion of DAM-like microglia expressing SPP1 increases with age, with this subtype being more prominent in the aged brain compared to the young brain (64). Following a stroke in juvenile mice, DAM-like microglia enter an irreversible state and are ultimately cleared from the brain. However, in newborn stroke models, the same DAM-like cells exhibit high plasticity, capable of reverting to a steady-state phenotype during recovery and reintegrating into the microglia network. This demonstrates that microglia possess stronger remodeling and repair capabilities during early development (84).

The specific heterogeneity of the microglia response shapes its unique disease trajectory. Therapeutic strategies targeting microglia in PD require precise intervention at specific microglial states during critical disease stages and within affected brain regions. Enhancing their protective phagocytic function may be beneficial in the early disease phase, whereas potent suppression of their harmful inflammatory responses may be necessary in the middle to late stages.

## 4 Genetic and gender heterogeneity in PD microglia

The clinical manifestations, progression rates, and treatment responses of PD exhibit significant heterogeneity among patients. Genetic background and gender are two fundamental intrinsic variables that influence PD susceptibility by regulating the functional state of microglia. There are innate differences in the immune response capacity of microglia between individuals (85). Gender influences the immune performance of microglia through sex chromosome gene dosage and lifelong regulation of sex hormones (86).

### 4.1 Genetic heterogeneity of microglia

PD patients exhibit a highly diverse genetic background, which directly influences microglia reactivity and the efficacy of therapeutic strategies. Genome-wide association studies (GWAS) have confirmed that variants at approximately ninety loci increase the risk of PD onset and progression (79). Over twenty distinct rare genetic variants constitute the familial PD genetic risk factors, leading to early-onset or atypical symptoms (85, 87). The discovery of genes associated with the progression and etiology of genetic PD, such as α-syn (88), Parkin (89), PINK1 (90), DJ-1 (91, 92), PARK82 (93), LRRK2, and FCGR2A (87), has advanced PD modeling and targeted therapeutic research. Genetic studies confirm that microglia significantly enrich risk variants for PD (**Figure 1F**) (94).

Genetic differences in microglial function between individuals are key determinants of PD susceptibility. LRRK2 is one of the most common mutation genes in both familial and sporadic PD (95, 96). Pathogenic mutations in the LRRK2 gene induce microglial-specific lysosomal dysfunction, impairing their clearance function and driving the progression of PD (97). Homozygous mutations in Glucocerebrosidase (GBA) are significant genetic risk factors for PD, with over 350 GBA1 variants accounting for 5–10% of PD cases (98, 99). The R47H mutation in TREM2 is a significant risk factor for PD (100). Genetic variants in TREM2-APOE directly impair the normal differentiation and function of microglia, weakening their phagocytic capacity and increasing susceptibility to PD (100–102). The genetic heterogeneity of microglia is also reflected in common risk genes across neurodegenerative diseases (103). Loss-of-function mutations in PARK2 and PARK6 encoding the E3 ubiquitin ligase Parkin and mitochondrial serine/threonine kinase PINK1 account for the majority of autosomal recessive early-onset PD cases. PARK2/PINK1 deficiency specifically exacerbates NLRP3 inflammasome overactivation and proinflammatory factor release in microglia (104). Major histocompatibility complex class II (MHC II)-associated gene SNPs constitute risk factors for PD (105). The genetic heterogeneity of PD influences the functional phenotype of microglia. Studies have constructed a subtype-centered microglial genomic atlas, isoMiGA, revealing through genetic analysis that PD genetic risk loci are significantly associated with the expression and splicing regulation of specific subtypes (106).

Genetic variations in PD patients carrying different gene mutations drive disease progression by impairing the phagocytic, degradative, and immunoregulatory functions of microglia. Given that the pathways and states of activated microglia in patients may exhibit fundamental differences, universal therapies may only be effective for specific genetic subpopulations. Therefore, therapeutic strategies targeting microglia must incorporate genetic biomarkers for patient stratification to achieve precision medicine for PD.

### 4.2 Gender heterogeneity in microglia

Gender is one of the risk factors for PD. Microglia respond to environmental challenges in a gender and time-dependent manner starting from the prenatal stage, exhibiting significant developmental and gender-dependent differences (**Figure 1G**) (19, 107). Gender exerts a significant influence on the gene expression, function, and inflammatory response of microglia, affecting multiple aspects, including their density, morphology, and phagocytic capacity (82). The relative risk of PD in male patients is twice that of women across all age groups (86). Compared with male patients, female patients have a higher risk of motor impairment in the early stages of the disease, slower decline in activities of daily living, and lower risk of cognitive impairment (108). Compared to female PD patients, male PD patients exhibit more severe cortical thinning in the central posterior and central anterior regions, along with smaller volumes in the thalamus, caudate nucleus, putamen, globus pallidus, hippocampus, and brainstem (109). The phagocytic capacity and intensity of inflammatory responses in male and female microglia may exhibit sex-dependent characteristics when responding to pathological stimuli (86). In PD animal models, male individuals exhibit stronger pro-inflammatory responses in microglia, leading to more severe dopaminergic neuron loss (86). Adult female mouse microglia exhibit a unique neuroprotective transcriptional phenotype. This protective phenotype persists when female microglia are transplanted into male brains, where they provide enhanced neuroprotection. Mechanistically, this reveals intrinsic gender heterogeneity in microglia, providing a crucial cellular immunological basis for gender differences in PD prevalence and clinical manifestations (110). Estrogen modulates the reactivity of microglia, inducing a protective phenotype in females, which is one of the key mechanisms underlying the reduced risk of PD onset in females (86). Genetic studies have also revealed no significant autosomal genetic differences across the entire genome between male and female PD patients, suggesting that sex chromosomes and environmental factors may be the primary drivers of PD’s gender heterogeneity (111).

The gender heterogeneity of microglia constitutes a key cellular basis for mediating sex differences in PD susceptibility, pathological progression, and clinical manifestations. Shaped by sex hormones, sex chromosome genes, and early developmental processes, male microglia tend to exhibit stronger pro-inflammatory properties in the context of PD, while female microglia retain greater neuroprotective potential. This suggests that interventions targeting PD should account for patient gender factors.

## 5 Therapeutic strategies targeting microglia

In PD treatment research, given the heterogeneity of microglial phenotypes, therapeutic strategies targeting microglia focus on modulating their polarization balance, encompassing multiple pathways including functional regulation, clearance of pathogenic factors, and remodeling of the immune microenvironment (112). Several clinical trials are currently underway to validate the feasibility and safety of targeting microglia for PD treatment (**Table 1**).

**Table 1:** Summary of clinical trials targeting microglia in Parkinson’s disease (data from clinicaltrials.gov).

Target category	Target	Mechanism/Strategy	Representative drug	Clinical study title	Intervention/Treatment	Mode of action	Clinical treatment effect	Phase	NCT number	Reference
Receptor	TLR2	Activated by pathological α-synuclein, triggering NLRP3 inflammasome assembly and pro-inflammatory responses.	NM-101	Single and Multiple Ascending Dose Study to Assess Safety, Tolerability and Pharmacokinetics of NM-101.	NM-101	anti-TLR2 antibody, Single and multiple ascending doses.	Research is ongoing.	Phase 1	NCT05790382	-
	TLR4	Trigger NLRP3 inflammasome assembly and regulate NF-κB.	Pentoxifylline	Role of Pentoxifylline and Celecoxib in Parkinsonism.	carbidopa-levodopa; Pentoxifylline 400 MG; Celecoxib 200mg	Inhibits the HMGB1-TLR4-NF-κB axis, suppressing microglial activation.	Research is ongoing.	Phase 2	NCT05962957	(144, 145)
	P2X7R	Key molecules in microglia activation and NLRP3 inflammasome activation.	P2X7R antagonist	P2X7 Receptor, Inflammation and Neurodegenerative Diseases (NeuroInfiam).	Memantine, Dopamine receptor-agonists	Observing the Protective Effects of P2X7 Purinergic Receptor (P2X7R) Antagonists in PD Microglia.	Research is ongoing.	Observational	NCT03918616	
	GLP-1	Agonists inhibit microglial activation.	Exenatide; NLY01; Lixisenatide; Liraglutide.	Exenatide Once Weekly Over 2 Years as a Potential Disease Modifying Treatment for Parkinson’s Disease (Exenatide-PD3)	Exenatide extended release 2mg (Bydureon)	Induce neurite outgrowth, promote neuronal differentiation, rescue degenerating neurons, and suppress microglial activation.	The drug is safe and well tolerated but has not shown significant therapeutic benefits in PD patients.	Phase 3	NCT04232969	(138, 139)
				Effects of Exenatide on Motor Function and the Brain	Exenatide, injection once per week for 1 year.	Inhibit microglia activation.	Research is ongoing.	Phase 1	NCT03456687	-
				Exenatide Treatment in Parkinson’s Disease	Exenatide Injections, 2 mg once weekly for 18 months	Increase neurogenesis, prevent or reverse substantia nigra-striatal damage, and activate GLP-1 receptors on microglia.	Research is ongoing.	Phase 2	NCT04305002	-
				A Clinical Study of NLY01 in Patient’s With Early Parkinson’s Disease	NLY01	Penetrates the blood-brain barrier, activates GLP-1 receptors on microglia, and exerts anti-inflammatory effects.	The drug is generally well-tolerated, but has not demonstrated any clinical therapeutic benefit.	Phase 2	NCT04154072	(137)
				Study to Evaluate the Effect of Lixisenatide in Patient With Parkinson’s Disease (LixiPark)	Lixisenatide (20 μg/d)	Increase neurogenesis and reduce microglial activation.	Effectively improved motor impairment in PD patients, but simultaneously caused side effects such as nausea and vomiting.	Phase 2	NCT03439943	(141)
				Safety and Efficacy of Liraglutide in Parkinson’s Disease	Liraglutide 6 mg/ml once daily at a maximum dose of 1.8 mg	Reduce activation of microglia and astrocytes.	Improved motor impairment in PD patients.	Phase 2	NCT02953665	(140)
Inflammasome	NLRP3 inflammasome	Inhibitors of NLRP3 inflammasomes disrupt inflammasome activation, suppress microglia activation, and prevent dopaminergic neuron loss.	VTX3232; VENT-02; HL-400; Dapansutrile.	Phase 2a Study of VTX3232 in Parkinson’s Disease	VTX3232	VTX3232 is an inhibitor of NLRP3, effectively suppressing NLRP3 and inhibiting downstream biomarkers, thereby suppressing microglial activation.	Research is ongoing.	Phase 2	NCT06556173	-
				A Phase 1b Study of the NLRP3 Inhibitor VENT-02 in Patients With Mild to Moderate Parkinson’s Disease	VENT-02	NLRP3 Inhibitor.	Research is ongoing.	Phase 1, Phase 2	NCT06822517	-
				First-in-Human Single and Multiple Dose of HL-400	HL-400	NLRP3 Inhibitor.	Research is ongoing.	Phase 1	NCT06997484	-
				A Trial to Test the Use of Dapansutrile, an Anti-inflammatory Medication, in People With Parkinson’s Disease (DAPA-PD)	Dapansutrile tablets administered for 26 weeks, starting at 1,000 mg daily (500 mg twice daily) for 4 weeks, escalated to 2,000 mg daily (1,000 mg twice daily) thereafter.	NLRP3 inhibitors prevent brain inflammation and protect against dopamine cell loss.	Research is ongoing.	Phase 2	NCT07157735	-
Signaling pathway	NF-κB pathway	Inhibition of the NF-κB pathway can alleviate neuroinflammation.	Cilostazol; Minocycline	Cilostazol in Parkinson Disease	Cilostazol	Inhibit NF-κB and downstream effectors (TNF-α and IL-1β), suppress the HMGB1/TLR4 axis.	Research is ongoing.	Phase 2	NCT06612593	-
				National Institute of Neurological Disorders and Stroke (NINDS) Parkinson’s Disease Neuroprotection Trial.	Minocycline	Inhibit the activation of the NF-κB signaling pathway to reduce microglial activation.	No clinical therapeutic benefits have been demonstrated.	Phase 2	NCT00063193	(142, 143)
Genetic risk factors	LRRK2	Inhibiting LRRK2 kinase enhances proteolytic function in microglial lysosomes, and strengthens the core protective function of microglial clearance of pathological proteins.	BIIB122; DNL151; DNL201; BIIB094; NEU-411.	Safety and Pharmacodynamic Effects of BIIB122 in Participants With LRRK2-Associated Parkinson’s Disease (LRRK2-PD)	BIIB122–225 mg	BIIB122 is an LRRK2 inhibitor that enhances the protective function of microglia in clearing cellular waste.	Research is ongoing.	Phase 2	NCT06602193	
				A Study to Learn About the Safety of BIIB122 Tablets and Whether They Can Slow the Worsening of Early-Stage Parkinson’s Disease in Adults Between the Ages of 30 and 80 (LUMA)	BIIB122–225 mg	Enhancing the protective function of microglia in processing cellular waste.	Research is ongoing.	Phase 2	NCT05348785	
				A Study to Evaluate the Safety, Tolerability, Pharmacokinetics, and Pharmacodynamics of DNL151 in Healthy Volunteers	DNL151	DNL151 is an LRRK2 inhibitor that enhances the protective function of microglia in processing cellular waste.	Safe and well-tolerated, effectively penetrates the central nervous system to inhibit peripheral and central LRRK2 kinase activity.	Phase 1	NCT04557800	(160)
				Study to Evaluate DNL151 in Subjects With Parkinson’s Disease	DNL151	DNL151 is an LRRK2 inhibitor that enhances the protective function of microglia in processing cellular waste.	Safe and well-tolerated, effectively penetrates the central nervous system to inhibit peripheral and central LRRK2 kinase activity.	Phase 1	NCT04056689	(160)
				Study to Evaluate DNL201 in Subjects With Parkinson’s Disease	DNL201	Inhibition of LRRK2 normalizes lysosomal function.	Reduce the levels of GSLs in the cerebrospinal fluid of LRRK2-PD patients	Phase 1	NCT03710707	(97)
				A Study to Evaluate the Safety, Tolerability, and Pharmacokinetics of BIIB094 in Adults With Parkinson’s Disease (REASON)	BIIB094	Inhibiting the excessive activity of LRRK2 kinase.	Research is ongoing.	Phase 1	NCT03976349	-
				A Phase 2 Study of NEU-411 in Companion Diagnostic-Positive Participants With Early Parkinson’s Disease (NEULARK)	NEU-411	NEU-411 is an orally administered, potent, selective, bioavailable, highly permeable, brain-penetrant, LRRK2-active small molecule inhibitor.	Research is ongoing.	Phase 2	NCT06680830	-
Enzyme	MPO	Verdiperstat (AZD3241) is the MPO inhibitor.	AZD3241	PET Study in Parkinson’s Disease Patients	AZD3241	Suppressing microglia activation and protecting dopaminergic neurons in PD patients.	Well tolerated.	Phase 2	NCT01527695	(118)

*Clinical trial data were retrieved from ClinicalTrials.gov; and published references are unavailable for some studies.

α-syn, Alpha-synuclein; BCise, Bydureon; GLP-1, Glucagon-like peptide-1; GSLs, Glycosphingolipids; HMGB1, High mobility group box 1; IL-1β, Interleukin-1 beta; LRRK2, Leucine-rich repeat kinase 2; MPO, Myeloperoxidase; NF-κB, Nuclear factor kappa-light-chain-enhancer of activated B cells; NOD-; NLRP3, LRR- and pyrin domain-containing protein 3; P2X7R, Purinergic receptor P2X 7; TLR2, Toll-like receptor 2; TLR4, Toll-like receptor 4; TNF-α, Tumor necrosis factor-alpha.

Directly suppressing harmful neuroinflammatory responses in microglia is a primary therapeutic target for PD. The NLRP3 inflammasome contributes to PD progression, making it a favored therapeutic strategy (113, 114). Multiple NLRP3 inhibitors, such as the clinically approved anti-inflammatory drug Rebamipide (Mucosta^®^) (115), MCC950 (116), and the endogenous inhibitor dopamine (117), effectively mitigate microglia-mediated PD neuroinflammation and adaptive immune cell infiltration, thereby protecting neurons and improving motor symptoms. VTX3232 effectively suppressed NLRP3 downstream biomarkers in a Phase 2a study for early-stage PD patients (NCT06556173). The safety and efficacy of inhibitors, including VENT-02, HL-400, and Dapansutrile for PD clinical trials, are still under validation (NCT06822517, NCT06997484, NCT07157735). The myeloperoxidase inhibitor AZD3241 suppresses microglia activation in PD patients, thereby protecting dopaminergic neurons, and demonstrated good tolerability in a Phase 2a trial (NCT01527695) (118). Blocking IL-6 or its downstream effects can suppress the disruption of neuronal homeostasis caused by α-syn-activated microglia (119). The specific inhibitor PAP-1 effectively suppresses the activity of Kv1.3 potassium channels in microglia, thereby mitigating microglia-mediated harmful reactions (120). Selective accumulation of α-syn within microglia leads to dopaminergic neuron degeneration in PD. Inhibiting the production of such toxic molecules protects neurons (121, 122). Knockout of macrophage antigen complex-1 (Mac1) or using its blocking peptide alleviates neuroinflammation via the NOX-NLRP3 axis (123). Osteoprotegerin (OPG) effectively alleviates neuroinflammation by modulating the RANK-RANKL-OPG axis in microglia to inhibit the NF-κB signaling pathway (124). Inhibition of NLRC5 protects dopaminergic neurons and improves motor function in PD by suppressing NF-κB and MAPK signaling pathways in PD microglia (125). Certain drugs, such as dextromethorphan (DXM) (126), VIP receptor agonists (127), silymarin (128), and the immunomodulator Lenalidomide (129), exert neuroprotective effects in PD by inhibiting microglial activation and the downstream release of inflammatory mediators (130).

In PD, α-syn-activated microglia promote the release of cathepsin L (CTSL) via the P2X7R/PI3K/AKT signaling pathway, leading to neuronal death. This process could be blocked by P2X7R antagonists or the exosome inhibitor GW4869 (131). Pathological α-syn fibrils (PFFs) bind to TLR2 receptors on the surface of microglia, initiating the downstream MyD88/NF-κB signaling pathway. This activates microglia to release large amounts of pro-inflammatory molecules, driving neuroinflammation. The therapeutic peptide wtTIDM and wtNBD effectively block the pro-inflammatory signaling cascade, suppressing the secretion of harmful inflammatory cytokines by microglia and achieving therapeutic effects in PD (132). A clinical study evaluated the safety, tolerability, and pharmacokinetics of the anti-TLR2 antibody NM-101 in PD patients (NCT05790382). In PD, microglia phagocytose α-syn via the TREM2 receptor, which is then cleaved by the asparaginyl endopeptidase (AEP) enzyme to produce toxic fragments that exacerbate pathological spread, for which inhibiting TREM2 or AEP may slow PD progression (133). The absence of the necrotic apoptosis executioner protein Mixed Lineage Kinase Domain-Like (MLKL) alleviates neuroinflammation by suppressing excessive activation of microglia in PD (134). GLP-1 agonists exert neuroprotective effects in PD by directly inhibiting microglial activation (135, 136). In clinical studies of PD, trials of NLY01 (NCT04154072) (137) and Exenatide (138, 139) (NCT04232969, NCT03456687, NCT04305002) did not demonstrate significant therapeutic benefits. Liraglutide (NCT02953665) (140) and Lixisenatide (NCT03439943) (141) effectively improved motor impairment in PD patients, but were associated with side effects of nausea and vomiting. The novel dual GLP-1/GIP receptor agonist DA-CH5 demonstrates superior efficacy in treating PD compared to Liraglutide, though its clinical effects require further validation (140). Minocycline reduces microglial activation by inhibiting NF-κB signaling pathway activation and demonstrates therapeutic effects in PD animal models. However, it failed to show significant therapeutic efficacy in a Phase II clinical trial (NCT00063193) (142, 143). Pentoxifylline suppresses microglial activation by inhibiting the HMGB1-TLR4-NF-κB axis, with ongoing clinical trials (NCT05962957, NCT06612593) (144, 145).

Reprogramming microglia from a harmful phenotype to a neuroprotective phenotype holds promise for halting the progression of PD. Multiple natural and synthetic compounds can regulate this process. Taurine inhibits the polarization of microglia toward the M1 phenotype, reduces the release of proinflammatory mediators, and protects dopaminergic neurons (146). Schisandrin (45) and Capsaicin (147) both induce the conversion of microglia to the M2 phenotype, thereby alleviating neuroinflammation in PD. Targeting specific receptors to drive microglia polarization toward a protective phenotype also represents a promising therapeutic strategy for PD. Activation of the TREM2 promotes phagocytosis and anti-inflammatory functions in microglia (148, 149). Dihydroquercetin (DHQ) suppresses neuroinflammation in PD and protects dopaminergic neurons by activating the TREM2 signaling pathway (150). Star-shaped microglial-derived IL-3 guides microglia to transition into a protective phenotype that enhances phagocytosis of α-syn, while simultaneously promoting neuronal autophagy (151). The lncRNA HOXA-AS2 promotes neuroinflammation in PD by regulating microglial polarization (152). Some innovative therapeutic approaches, such as the integrated nanoreactor (Q@CeBG), can penetrate the blood-brain barrier and reprogram microglia from the M1 to the M2 phenotype (153). Self-catalytic small interfering RNA (siRNA) S/Ce-PABMS nanocarriers alleviate neuroinflammation by indirectly calming microglia from a harmful hyperactivated state through reducing α-syn aggregation (154). Modulating the gut microbiota can also regulate the state of microglia, offering insights into controlling PD neuroinflammation by intervening in the gut-brain axis (155).

Enhancing the intrinsic protective functions of microglia, particularly their ability to clear pathological proteins, represents another key therapeutic direction for PD. Complete remodeling of the microglia population using PLX3397 induces a neuroprotective phenotype that resists dopaminergic damage (156). Core-shell IHM nanoparticles can penetrate the brain to reprogram diseased microglia, degrade α-syn, while neutralizing neuroinflammatory factor (43). The two immunoglobulin-like (Ig-like) domains of α-syn receptor activate microglia to release proinflammatory factors. Ig-like binders can effectively block α-syn aggregation and reduce the inflammatory response of microglia (157). Enhancing the phagocytic capacity of microglia themselves is a key approach to inducing a protective phenotype. The natural small molecule kaempferol (Ka) selectively degrades NLRP3 protein by promoting autophagy in microglia (113). Multiple LRRK2 kinase inhibitors have demonstrated therapeutic potential for treating microglia lysosomal dysfunction caused by LRRK2 gene mutations (158, 159). ARV-102 reduced biomarkers of lysosomal and neuroinflammatory microglial pathways in a Phase I clinical trial, demonstrating positive tolerability (the trial was conducted under the supervision of the European Medicines Agency (EMA), with EUCT number 2024-516888-84-00). Two early-phase clinical studies demonstrated that BIIB122 was safe and well-tolerated over a 28-day treatment period. It effectively penetrated the central nervous system, exhibited dose-dependent inhibition of LRRK2 kinase activity, and modulated downstream biomarkers. However, its therapeutic efficacy for PD remains unclear and requires validation through larger-scale late-phase clinical trials (NCT04557800, NCT04056689) (160). A clinical trial of BIIB122 (DNL151) is ongoing (NCT06602193). DNL201 reduces levels of gangliosides (GSL) in the cerebrospinal fluid of LRRK2-PD patients (NCT03710707) (97). Additionally, clinical trials such as BIIB094, NEU-411, and DNL151 are currently underway (NCT03976349, NCT06680830, NCT04056689).

Therapeutic strategies targeting microglia in PD primarily focus on suppressing their neurotoxicity while preserving or enhancing their neuroprotective functions. In the complex internal environment, drug interventions may produce unpredictable network effects. Achieving balanced regulation of therapeutic regimens represents a major challenge for targeted microglia therapy in PD. Research on non-pharmacological interventions such as exercise intervention (NCT06993142) and intermittent fasting for slowing the progression of early-stage PD is advancing, providing important complementary options to existing treatment regimens for PD (161).

## 6 Discussion

In recent years, the clinical diagnostic criteria for PD have been refined, research standards for the prodromal phase have been introduced, and numerous genetic risk loci have been identified. These advances are transforming PD from a purely clinically diagnosed entity into a disease entity supported by biomarkers, enabling early identification and differentiation of distinct prognostic subtypes (162). Microglia, as the core immune regulators of the central nervous system, have become a research focus due to the high heterogeneity of their functions and states (163). Dynamic and heterogeneous microglia in the brain exhibit multidimensional activation states in PD, performing multiple roles, including neuroprotective functions such as clearing pathological proteins and promoting repair, along with excessive activation, inducing neuroinflammation. They serve as regulators of neuronal function and homeostasis (164, 165). Significant variations in clinical manifestations among individual patients correlate with distinct response patterns of microglia (166).

PD microglia exhibit heterogeneity across multiple dimensions, including functional, spatiotemporal, genetic, and gender aspects. Middle-aged and elderly individuals constitute the high-risk population for PD. Aging microglia exhibit reduced clearance efficiency and secrete large amounts of pro-inflammatory factors, forming a vicious cycle with genetic risks such as LRRK2 and TREM2, as well as region-specific susceptibility, aggravating PD pathology (167). Targeting microglia offers a promising avenue for treating neuroinflammation and neurodegenerative diseases by unlocking the full potential of microglia as therapeutic targets to seek effective treatments for central nervous system disorders. Although therapeutic strategies targeting microglia have demonstrated significant potential in preclinical studies, their translation to clinical settings faces major challenges. These include the highly dynamic and reactive nature of microglia, the inability of single animal models to fully replicate the pathological features of human PD, and substantial interindividual heterogeneity (54, 168). Bridging these gaps will enhance our understanding of the evolutionary diversity, tissue specificity, and PD-related significance of the microglia lineage. To achieve precise targeting of microglia in PD patients, future research should pursue in-depth exploration and breakthroughs across multiple fronts. Integrating genetic background (e.g., LRRK2 and GBA mutation status), disease staging (based on biomarkers), microglia activity (based on PET imaging), and factors such as age and gender to construct personalized treatment models facilitates precision medicine for PD.

In summary, the heterogeneity of microglia represents a central thread running through the development and progression of PD. Delving into the heterogeneity of microglia will lay the groundwork for establishing a new paradigm in precision immunotherapy for PD. Interventions targeting microglia phenotype acquisition and immune dysfunction in PD high-risk patients may emerge as novel therapeutic strategies for early intervention and disease improvement. Through in-depth understanding and exploration of microglia heterogeneity, we can unlock the immense potential of microglia to deliver truly effective personalized disease-modifying therapies for PD patients.
